# Erratum - A Novel Model for Studying Voltage-Gated Ion Channel Gene Expression during Reversible Ischemic Stroke

**DOI:** 10.7150/ijms.39498

**Published:** 2019-09-18

**Authors:** Yun-Shen Tai, Shih-Chieh Yang, Yi-Chun Hsieh, Yaw-Bin Huang, Pao-Chu Wu, Ming-Jun Tsai, Yi-Hung Tsai, Ming-Wei Lin

**Affiliations:** 1 Department of Surgery, E-Da Hospital, Kaohsiung, Taiwan; 2 Department of Orthopedic Surgery, E-Da Hospital, Kaohsiung, Taiwan; 3 Department of Anesthesiology, University of Maryland School of Medicine, Baltimore, MD, USA.; 4 School of Pharmacy, Kaohsiung Medical University, Kaohsiung, Taiwan; 5 Center for Stem Cell Research, Kaohsiung Medical University, Kaohsiung, Taiwan; 6 Department of Neurology, China Medical University Hospital, Taichung, Taiwan; 7 School of Medicine, China Medical University, Taichung, Taiwan; 8 Department of Neurology, China Medical University, An-Nan Hospital, Tainan, Taiwan; 9 Department of Medical Research, E-Da Hospital/ E-Da Cancer Hospital, Kaohsiung, Taiwan; 10 I-Shou University College of Medicine, Kaohsiung, Taiwan

The first author's affiliation should be corrected by adding “I-Shou University College of Medicine, Kaohsiung, Taiwan” (Affiliation No. 10), to the “Department of Surgery, E-Da Hospital, Kaohsiung, Taiwan.” (Affiliation No. 1), as shown in the title page above.
